# Recycling Microplastics to Fabricate Anodes for Lithium‐Ion Batteries: From Removal of Environmental Troubles via Electrocoagulation to Useful Resources

**DOI:** 10.1002/advs.202205675

**Published:** 2023-01-16

**Authors:** Jinhee Lee, Yong‐Tae Kim, Jinsub Choi

**Affiliations:** ^1^ Department of Chemistry and Chemical Engineering Inha University Incheon 22212 Republic of Korea

**Keywords:** electrocoagulation, iron oxide, lithium‐ion batteries, microplastics, waste recycling

## Abstract

Electrocoagulation is an evolving technology for the abatement of a broad range of pollutants in wastewater owing to its flexibility, easy setup, and eco‐friendly nature. Here, environment‐friendly strategies for the separation, retreatment, and utilization of microplastics via electrocoagulation are investigated. The findings show that the flocs generated by forming Fe_3_O_4_ on the surface of polyethylene (PE) particles are easily separated using a magnetic force with high efficiency of 98.4%. In the photodegradation of the obtained flocs, it is confirmed that Fe_3_O_4_ shall be removed for the efficient generation of free radicals, leading to the highly efficient photolysis of PE. The removed Fe_3_O_4_ can be recycled into iron‐oxalate compounds, which can be used in battery applications. In addition, it is suggested that heat treatment of Fe_3_O_4_–PE flocs in an Ar atmosphere leads to forming Fe_3_O_4_ core–carbon shell nanoparticles, which show excellent performance as anodes in lithium‐ion batteries. The proposed composite exhibits an excellent capacity of 1123 mAh g^−1^ at the current density of 0.5 A g^−1^ after 600 cycles with a negative fading phenomenon. This study offers insight into a new paradigm of recyclable processes, from environmental issues such as microplastics to using energy materials.

## Graphical abstract

As a sustainable and environmental strategy, microplastics decorated with iron oxide nanoparticles are effectively collected and separated from the media for the application as anode materials with high specific capacity and stability via appropriate post‐treatment process, opening a new paradigm of recyclable from environmental issues to energy applications.

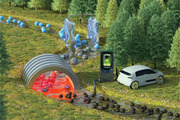

## 1 Introduction

Plastics are artificially manufactured organic polymers that can be fabricated into various shapes. Typically, plastics are affordable, lightweight, have remarkable plasticity and flexibility, high thermal and electrical insulation, and corrosion resistance.^[^ ^1^ ^]^ Owing to these superior properties and stability, the use of plastics has exploded since the beginning of industrial‐scale plastic production in the 1950s.^[^ ^2^ ^]^ The annual production of fossil‐fuel‐based plastics is expected to exceed 1.2 billion tons, and waste is expected to exceed 1 billion tons by 2060.^[^ ^3^ ^]^ The flow of “raw material extraction, synthesis, use, and disposal” in the plastic industry is a prime example of a linear economy that inevitably causes environmental pollution.^[^ ^4^, ^5^ ^]^ Generally, microplastics with diameters smaller than 5 mm can be classified into primary microplastics, designed as tiny particles for commercial use, and secondary microplastics, resulting from the breakdown of larger plastic items caused by exposure to environmental factors such as ultraviolet (UV) irradiation and ocean waves.^[^ ^6^, ^7^ ^]^ Both classes of microplastics have received considerable attention as emerging contaminants, owing to their potential ecological risks.^[^ ^8^, ^9^ ^]^ For example, microplastics can transport toxic organic chemicals owing to their high specific surface area and strong hydrophobic properties. Furthermore, numerous heavy metals, including zinc, copper (Cu), lead, and silver, can be adsorbed onto the surface of microplastics.^[^ ^10^, ^11^, ^12^ ^]^ Microplastics are not readily broken down into harmless molecules in oceans; they take hundreds or thousands of years to decompose.^[^ ^13^ ^]^ Instead, marine animals often consume them, thus affecting ecosystems and human health through the food chain.^[^ ^14^ ^]^ To solve such environmental problems posed by microplastics that threaten the future of humanity, it is necessary to transform the linear economy of microplastics into a circular economy through the collection and reuse/recycling of aggregates by appropriate approaches to prevent secondary pollution.^[^ ^5^, ^15^ ^]^

Microplastics are commonly removed using an anaerobic reactor or an aerobic membrane reactor, and by various approaches like electrooxidation, microfiltration, chemical coagulation, and photocatalytic ozonation.^[^ ^16^ ^]^ Electrocoagulation is an electrochemical approach that provides a simple, rapid, and cost‐effective method for removing pollutants suspended in an aqueous medium without using any added chemicals. During the electrocoagulation process, metal (commonly Al and Fe) ions are produced at the anode under an electric field, which then react with hydroxide ions, combine with the microplastic, sink together by colliding flocs, and neutralize the oppositely charged suspended colloidal particles.^[^ ^17^, ^18^ ^]^ Anode materials, types and shapes of microplastics, and concentration of microplastics in wastewater all affect the removal of microplastics by electrocoagulation.^[^ ^19^ ^]^ Perren et al. evaluated the removal of 300 µm spherical polyethylene (PE) microbeads using an Al electrode under different pH conditions and current densities. Sodium chloride was added for process performance improvement, achieving a removal efficiency that ranged from 90% to 99%, showing great potential for removing microplastics from wastewater.^[^ ^20^ ^]^ Furthermore, Shen et al. demonstrated that a system with an Al anode has a better removal effect for microplastics than a process using Fe anodes. They also showed that the efficiency of electrocoagulation‐based removal of fiber microplastics was superior to that of granular microplastics.^[^ ^17^ ^]^

In this study, we designed and investigated an electrocoagulation–magnetic separation process using Fe metal as a sacrificial electrode, where Fe_3_O_4_ acts as a coagulant to neutralize and separate microplastics to form flocs with magnetic properties. The mechanism of the electrocoagulation reaction, possibility of magnetic separation, and application of the treated waste were studied by targeting PE as a representative material for microplastics. Fe_3_O_4_ adsorbed on the surfaces of the flocs interferes with electron–hole pair formation, hampering the photolysis of plastics. In addition, the application of Fe_3_O_4_ as an anode for lithium‐ion batteries (LIBs) is examined. Fe_3_O_4_ is regarded as one of the promising candidates to replace the commercial graphite anode owing to its ability to store up to eight Li^+^ per formular unit leading to a high theoretical specific capacity of 924 mAh g^−1^. However, several drawbacks, including poor capacity retention, low Li^+^ diffusion coefficient, and large volume change during charging/discharging with low electronic conductivity led to reduced cycle stability and poor rate performance. Carbon coatings have been extensively studied to alleviate the mass change problem of transition metal oxides and increase their electronic conductivity. Notably, in this study, the Fe_3_O_4_ core–carbon shell nanoparticles were obtained by converting the entrapped plastic surrounding Fe_3_O_4_ into a carbon layer in an Ar atmosphere. The prepared electrode contributes to the formation of stable solid electrolyte interface (SEI) layer, alleviating pulverization caused by the volume expansion of transition‐metal‐based electrode, and enhancing electrical conductivity. This process afforded an excellent capacity of 1123 mAh g^−1^ at the current density of 0.5 A g^−1^ after 600 cycles with a negative fading phenomenon, mainly induced by the decomposition of the electrolyte‐derived surface layer. These results can provide insights into circular strategies for effectively removing microplastics that cause environmental problems and utilizing waste after suitable post‐treatment in energy applications.

## 2 Results and Discussion

### 2.1 Electrocoagulation of PE

**Scheme 1:**
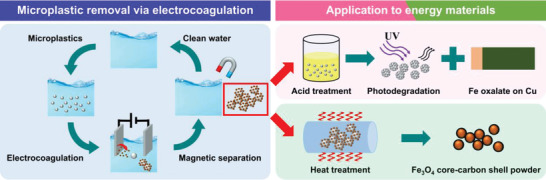
Schematic diagram of microplastic removal process and application of waste flocs in energy materials.

**Figure 1:**
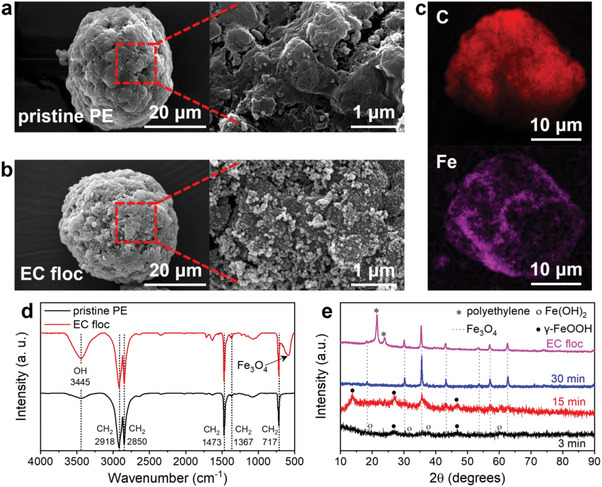
Scanning electron microscopy (SEM) images of a) pristine PE and b) Fe_3_O_4_–PE floc. c) Mapping images of Fe_3_O_4_–PE floc. d) Fourier‐transform infrared (FT‐IR) spectra of pristine PE and Fe_3_O_4_–PE floc, demonstrating that PE is captured by electrocoagulation. e) X‐ray diffraction (XRD) patterns of flocs in different reaction times imply that the phase of floc is changing during electrocoagulation.

**Scheme** **1** shows the configuration for separating the PE from the suspended solution through electrocoagulation. Electrocoagulation removes pollutants suspended in an aqueous medium by destabilizing the repulsive force between particles by applying an electric current. Electrodes made of consumable metals like Al and Fe, called sacrificial electrodes, continuously form metal ions by oxidation, and metal cations and metal hydroxides play a role in neutralizing suspended particles during the electrocoagulation process.^[^ ^21^ ^]^ The scanning electron microscopy (SEM) images show the morphology of pristine PE before (**Figure** **1**a) and after the electrocoagulation process with mapping images (Figure 1b,c). There is no noticeable change in the overall size of the PE particle. However, it can be seen that nanosized particles composed of iron oxide were formed and completely covered on the surface of the PE particle, resulting in the destabilized suspended PE particles for easy separation from the medium.^[^ ^22^ ^]^ Fourier‐transform infrared (FT‐IR) spectra (Figure 1d) indicate that the floc had a component peak identical to that of pristine PE, with additional Fe_3_O_4_ peaks^[^ ^23^ ^]^ derived from nanoparticles formed on the surface of the PE particles.

**Figure 2:**
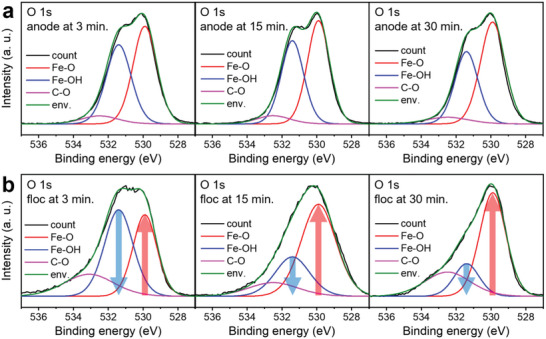
X‐ray photoelectron spectroscopy (XPS) results: O 1s spectra of a) anode surface and b) collected flocs in different reaction times.

The X‐ray diffraction (XRD) spectra recorded at different reaction times were investigated to determine the floc formation mechanism (Figure 1e). In the initial stage of electrocoagulation (3 min), Fe(OH)_2_ (JCPDS 13‐0089) was formed via the hydrolysis of Fe^2+^ produced by electrolysis of the Fe electrode.^[^ ^24^ ^]^ After 15 min, *γ*‐FeOOH (JCPDS 44‐1415) and Fe_3_O_4_ (dotted line, JCPDS 19‐0629) peaks appeared, while the Fe(OH)_2_ peak diminished in intensity. In the final stage of electrocoagulation (30 min), only the Fe_3_O_4_ peaks remained, demonstrating that the initially formed Fe(OH)_2_ was transformed to Fe_3_O_4_ through the *γ*‐FeOOH intermediate. This outcome can also be confirmed by the color change of the solution (green to orange to brown) as electrocoagulation proceeded (Figure S1, Supporting Information). The chemical states of the Fe anode surfaces and flocs obtained at different electrocoagulation stages were analyzed by X‐ray photoelectron spectroscopy (XPS) for further investigation. The O 1s spectra of the Fe anode maintained a strong Fe—OH peak (531.4 eV) throughout the entire reaction period (**Figure** **2**a), while the peak intensity of the Fe—OH peak in flocs diminished continuously with the enhanced peak intensity of Fe—O as the electrocoagulation proceeded (Figure 2b). The chemical shift to lower binding energy in the Fe 2p spectra also indicated the transition of the state from oxyhydroxide to oxide with a reduced degree of oxidation^[^ ^25^ ^]^ (Figure S2, Supporting Information). These results imply that Fe(OH)_2_ was formed at the surface of the anode electrode by the reaction of dissolved Fe^2+^ cations with hydroxide ions detached and transformed into Fe_3_O_4_, which served as a coagulant for PE removal through the following mechanisms (Equations ((1), (2), (3), (4), (5)))

(1) $$\mathrm{Fe}\left(s\right)\to {\mathrm{Fe}}^{2+}\left(\mathrm{aq}\right)+2e^{-}\left(\mathrm{aq}\right)$$

(2) $$2H_{2}O\left(l\right)+2e^{-}\left(\mathrm{aq}\right)\to H_{2}\left(g\right)+2{\mathrm{OH}}^{-}\left(\mathrm{aq}\right)$$

(3) $${\mathrm{Fe}}^{2+}\left(\mathrm{aq}\right)+2{\mathrm{OH}}^{-}\left(\mathrm{aq}\right)\to \mathrm{Fe}{\left(\mathrm{OH}\right)}_{2}\left(s\right)$$

(4) $$2\mathrm{Fe}{\left(\mathrm{OH}\right)}_{2}\left(s\right)+\frac{1}{2}O_{2}\left(g\right)\to 2\mathrm{FeOOH}\left(s\right)+H_{2}O\left(l\right)$$

(5) $$2\mathrm{FeOOH}\left(s\right)+\mathrm{Fe}{\left(\mathrm{OH}\right)}_{2}\left(s\right)\to {\mathrm{Fe}}_{3}O_{4}\left(s\right)+2H_{2}O\left(l\right)$$

At the beginning of electrocoagulation, Equations (1) and (2) occur in pairs at the anode and cathode, respectively. The pH of the electrolyte changed from neutral to alkaline owing to hydrogen evolution (Figure S3, Supporting Information), resulting in the precipitation of Fe(OH)_2_. Owing to the thermodynamic instability of Fe(OH)_2_, it is immediately transformed into Fe_3_O_4_ via the FeOOH intermediate (Equations (4) and (5)).^[^ ^26^ ^]^

**Figure 3:**
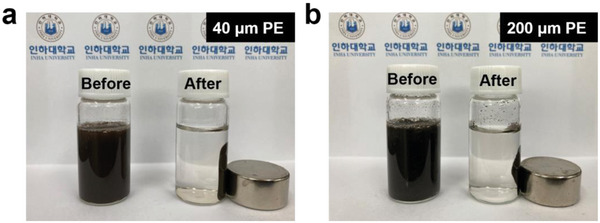
Conceptual photographs of magnetic separation using an external magnet. a) Flocs from PE 40 µm in diameter, and b) flocs from PE 40 µm in diameter.

Because the Fe_3_O_4_ that covers the surface of PE has magnetic properties, Fe_3_O_4_–PE flocs could be directly separated from the suspended solution by applying a magnetic field (**Figure** **3**a,b and Video S1 (Supporting Information)). It was confirmed that ≈0.492 g out of the 0.5 g of PE could be collected by electrocoagulation and magnetic separation techniques, indicating that the removal efficiency is ≈98.4% (**Table** **1**).

**Table 1:** Measured weight data obtained by repeating each experiment 5 times for calculation of removal efficiency

Flocs	Weight [g]					Average weight [g]	PE flocs^a)^ [g]	Efficiency^b)^ [%]
	1	2	3	4	5			
Fe_3_O_4_–PE flocs	0.523	0.529	0.524	0.527	0.527	0.526	0.492	98.4
Fe_3_O_4_ flocs	0.033	0.032	0.034	0.034	0.035	0.034	–	–

^a)^ PE flocs = (average of Fe_3_O_4_–PE flocs) − (average of Fe_3_O_4_ flocs);

^b)^ $\mathrm{Efficiency}=\frac{\mathrm{PE}\;\mathrm{flocs}}{{\mathrm{PE}}_{0}}\times 100\left[\%\right]$, PE_0_ = initial amount of polyethylene, 0.5 g.

Similar experiments were carried out by changing the size of PE or the concentration of the NaCl solution to verify whether electrocoagulation occurs equally in the other conditions. Comparing Figure 1b and Figure S4a (Supporting Information), the density of nanosized particles adsorbed on the surface of PE decreased as the size of PE increased from 40 to 200 µm. This outcome may be attributed to the low mobility of PE particles, leading to a reduced opportunity of colliding with Fe_3_O_4_ particles (Figure S4a, Supporting Information). However, there was no deterioration in the separation of PE or removal efficiency using a magnet, indicating that this approach was still effective in removing suspended microplastics larger than 200 µm. In addition, when the concentration of NaCl solution increasing from 0.4 to 3.5 wt% (like seawater) was used with 40 µm sized PE, there were no differences in morphologies and compositions of flocs (Figure 1b and Figure S4b (Supporting Information)). However, there was a notable difference in the overpotential: 2.4 and 1.3 V for 0.4 and 3.5 wt% NaCl, respectively (Figure S4c, Supporting Information). Therefore, it is reasonable to assume that the separation of microplastics in seawater can progress with low energy consumption without additional additives, considering only salinity and no impurities. In addition to sodium chloride, real seawater contains various cations such as magnesium, calcium, potassium, and strontium, and anions such as sulfate, borate, carbonate, and fluoride, so further research is needed on how these impurities affect the electrocoagulation reaction.

### 2.2 Photodegradation of Fe_3_O_4_–PE Flocs

**Figure 4:**
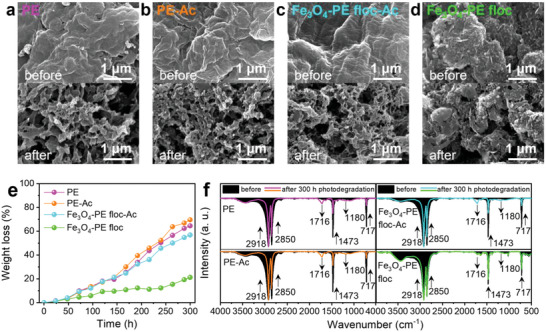
SEM images of a) PE, b) PE–Ac, c) Fe_3_O_4_–PE floc–Ac, d) Fe_3_O_4_–PE floc before and after photodegradation test. e) Weight loss profiles of each sample during photodegradation for 300 h. f) FT‐IR spectra of each sample. The black area denotes FT‐IR spectra of the samples before degradation, and the colored lines denote the spectra after the photodegradation test.

Photodegradation induced by UV light irradiation is one of the leading causes of non‐biological aging of microplastics.^[^ ^27^ ^]^ PE and Fe_3_O_4_–PE flocs were prepared to investigate the effect of Fe_3_O_4_ adsorbed onto the surface of PE on the photodegradation. In addition, oxalic acid treatment was carried out to remove Fe_3_O_4_ from the PE flocs, and all samples were prepared to have a uniform thickness of 40 µm on a slide glass using the doctor blade method (Figure S5, Supporting Information). The samples are denoted as PE, PE–Ac, Fe_3_O_4_–PE floc, and Fe_3_O_4_–PE floc–Ac for clarity, where Ac stands for oxalic acid treatment. **Figure** **4**a–d shows the SEM images of the samples before and after UV irradiation to observe the degree of photoaging. All the samples exhibited a smooth and homogeneous surface morphology before UV irradiation. However, cracks and pits were observed on the sample surface after UV irradiation, except for the Fe_3_O_4_–PE flocs. Consistent with the SEM images, Figure 4e shows that the weight losses of PE, PE–Ac, and Fe_3_O_4_–PE floc–Ac under UV irradiation for 300 h were 64.53%, 69.49%, and 56.88%, respectively. Meanwhile, the weight loss of the Fe_3_O_4_–PE floc was only 21.24%. This result strongly indicates that the acid treatment did not affect photolysis, but the presence of Fe_3_O_4_ inhibited the photolysis of PE.

Figure 4f shows the changes in the surface functional groups of the samples before and after the UV irradiation. For PE, PE–Ac, and Fe_3_O_4_–PE floc–Ac, the characteristic peak PE intensities at 2918, 2850, 1473, and 717 cm^−1^ correspond to C—C asymmetric stretching, symmetric stretching, scissoring, and rocking vibrations,^[^ ^28^, ^29^ ^]^ respectively. The peaks mentioned above decreased discernibly as the C=O stretching peak at 1716 cm^−1^ and C—O asymmetric stretching vibration peak at 1180 cm^−1^ appeared.^[^ ^28^, ^30^, ^31^ ^]^ The decrease in the C—C peak intensity and appearance of C=O and C—O peaks are evidence of random chain scission and photooxidation, suggesting that PE was degraded by UV irradiation.^[^ ^32^ ^]^ However, the Fe_3_O_4_–PE floc showed no discernible changes, such as a decrease in peak intensity or the appearance of new peaks owing to the existence of Fe_3_O_4_ inhibiting photolysis. The carbonyl index (CI) between the integrated area of 1850–1650 and 1500–1420 cm^−1^ in FT‐IR is another indicator used to quantitatively evaluate the aging of PE during photodegradation.^[^ ^33^ ^]^ The CI of PE, PE–Ac, and Fe_3_O_4_–PE floc–Ac was ≈1.5, which was higher than that of the Fe_3_O_4_–PE floc (0.413) (Table S1, Supporting Information). The low photodegradation efficiency of the sample containing Fe_3_O_4_ is attributed to the low bandgap of Fe_3_O_4_ (0.1 eV).^[^ ^34^ ^]^ Photodegradation is generally initiated in the presence of free radicals produced by electron–hole pairs, which are excited by irradiated photons.^[^ ^32^, ^35^ ^]^ However, low‐bandgap Fe_3_O_4_ becomes the recombination center of photogenerated electron–hole pairs, resulting in retarded photolysis of PE.

Interestingly, the iron species dissolved in oxalic acid from the Fe_3_O_4_–PE flocs can be reused via electroplating on a Cu substrate. SEM images (Figure S6a, Supporting Information) and XRD analysis (Figure S6b, Supporting Information) indicate that the electrochemically deposited material from iron‐oxide‐dissolved oxalic acid was FeC_2_O_4_•2H_2_O (JCPDS 22‐0635), which is one of the promising anode materials for next‐generation lithium‐ion batteries with high specific capacity^[^ ^36^, ^37^ ^]^ (Figure S6c, Supporting Information).

### 2.3 Application of Flocs as Anode Material for Lithium‐Ion Battery

Fe_3_O_4_–PE flocs, a type of solid waste produced in electrocoagulation to remove microplastics suspended in the medium, are detrimental to the environment and human health. Traditional disposal methods for flocs, such as landfills and incineration, can cause secondary pollution by leaching, diffusion, and resuspension in the environment; therefore, an effective stabilization treatment is highly recommended.^[^ ^38^ ^]^ Fe_3_O_4_ adsorbed on the surface of PE in flocs has been studied as a promising anode material for LIBs because of its ability to react with multiple Li^+^ ions per formula unit, enabling a high theoretical capacity (927 mAh g^−1^), high operating voltage (3.0 V), and eco‐friendliness.^[^ ^39^ ^]^ Furthermore, the large amount of carbon originating from PE can be applied as a source of conductive material to alleviate the low electrical conductivity of Fe_3_O_4_,^[^ ^40^, ^41^ ^]^ which is considered one of the main disadvantages of transition‐metal‐oxide‐based anode materials.

**Figure 5:**
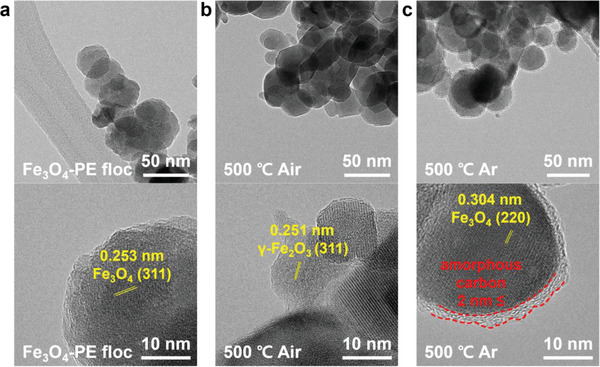
Field‐emission transmission electron microscopy (FE‐TEM) images of a) Fe_3_O_4_–PE floc, b) Fe_3_O_4_–PE floc treated at 500 °C in air, and c) Fe_3_O_4_–PE floc treated at 500 °C in Ar atmosphere. The bottom TEM images are enlarged views.

A single‐step heat treatment was applied at 500 °C for 2 h under air or Ar atmospheres to investigate the effect of the annealing atmosphere on the morphologies and compositions of the active materials derived from the Fe_3_O_4_–PE flocs. Figure S7a (Supporting Information) shows the XRD spectra of the Fe_3_O_4_–PE flocs after heat treatment in different atmospheres. It was confirmed that the magnetite present in the Fe_3_O_4_–PE flocs was converted into a mixed phase of maghemite and hematite during heat treatment in air, whereas the magnetite annealed in Ar maintained its composition even after heat treatment. Figure S7b (Supporting Information) shows that most of the carbon components disappeared in the temperature range of 400–500 °C, regardless of the heat treatment atmosphere, leaving only ≈4.46–4.6% of the iron oxide composites. The changes in the morphologies before and after heat treatment in different atmospheres were investigated by transmission electron microscopy (TEM) analysis. The TEM image of the Fe_3_O_4_–PE flocs (**Figure** **5**a) shows that spherical particles with a uniform diameter (≈50 nm) were prepared during the electrocoagulation process. The high‐resolution TEM image reveals lattice fringes with a *d*‐spacing of 0.253 nm, consistent with the *d* value for the (311) plane of Fe_3_O_4_. After heat treatment in the air (Figure 5b), although there was no remarkable change in the morphology, the interplanar distance was reduced to 0.251 nm, corresponding to the (311) plane of *γ*‐Fe_2_O_3_, consistent with the XRD analysis. However, the magnetite component on the heat‐treated Fe_3_O_4_–PE flocs had an amorphous carbon layer covering the surface of the oxide nanoparticles with a uniform thickness (<2 nm) (Figure 5c). This covering formed an oxide core–carbon shell structure (Figure S8, Supporting Information). The magnetite component has a lattice fringe with a *d*‐spacing of 0.304 nm corresponding to the (220) plane of magnetite.^[^ ^42^ ^]^ These results imply that the organic compound, i.e., PE, became conductive carbon shells after annealing in Ar. The weight ratio of Fe_3_O_4_:C of the Fe_3_O_4_–PE flocs after heat treatment in Ar, as measured by thermal gravimetric analysis (TGA), is 10.47:1 (Figure S9, Supporting Information). The intact shell serves as a buffering layer in oxide core–carbon shell structures which can mitigate the volume expansion of Fe_3_O_4_. This layer is considered one of the most significant drawbacks of transition‐metal‐oxide‐based anode materials.^[^ ^43^ ^]^ Furthermore, the buffering layer blocks direct contact between the active material and electrolyte that contributes to forming a stable SEI film, resulting in long‐term cycling stability and increased electron conductivity for fast Li‐ion diffusion.^[^ ^44^ ^]^

Fe_3_O_4_–PE flocs were annealed at 500 °C with a ramping rate of 5 °C min^−1^ for 2 h under air or Ar atmospheres. Then, the heat‐treated floc and graphite were mixed in a weight ratio of 3:7 to prepare an active material denoted as g–floc–air and g–floc–Ar, respectively. The CV curves of graphite, g–floc–air, and g–floc–Ar are analyzed in Figure S10a–c (Supporting Information) to confirm that the active material prepared by electrocoagulation and subsequent heat treatment of PE to be used as an anode electrode for LIBs. The reduction peaks at about 0.75 and 0.1 V appeared during the first cathodic scan for graphite, corresponding to the formation of the SEI layer and the intercalation of Li ions into graphite, respectively. The corresponding anodic peak shown at 0.25 V stemmed from the deintercalation of Li ions from the graphite electrode.^[^ ^45^ ^]^ The reduction peak at 0.75 V disappeared in subsequent cycles, revealing that the SEI formation process occurs predominantly in the first cycle.^[^ ^46^ ^]^ For g–floc–air and g–floc–Ar, the peak at 0.84 V was ascribed to the formation of Li*_x_* Fe_3_O_4_ resulting from the insertion of Li ions (Equation (6)). Another peak at 0.64 V is associated with the reduction of Li*_x_* Fe_3_O_4_ to Fe and the formation of SEI mainly composed of Li_2_O according to Equation (7) that follows

(6) $${\mathrm{Fe}}_{3}O_{4}+x{\mathrm{Li}}^{+}+xe^{-}\to {\mathrm{Li}}_{x}{\mathrm{Fe}}_{3}O_{4}$$

(7) $$\left(8-x\right){\mathrm{Li}}^{+}+{\mathrm{Li}}_{x}{\mathrm{Fe}}_{3}O_{4}+\left(8-x\right)e^{-}\to 4{\mathrm{Li}}_{2}O+3{\mathrm{Fe}}^{0}$$

In the subsequent anodic sweep, the peaks at 1.61 and 1.85 V are attributed to the oxidation of Fe to Fe^2+^ and Fe^3+^, respectively.^[^ ^47^, ^48^ ^]^ Interestingly, the peak of g–floc–Ar at 0.64 V, associated with the formation of the SEI layer, is noticeably more intense than that of g–floc–air. The irreversible capacity loss of g–floc–Ar (29.28%) was higher than that of g–floc–air (21.56%) (Figure S10d, Supporting Information). This result may be due to the difference in the surface area of the iron oxide nanoparticles, which was confirmed by Brunauer–Emmett–Teller analysis (Table S2, Supporting Information). In general, the surface area of the nanoparticles decreases because agglomeration occurs during the heat treatment.^[^ ^49^ ^]^ For g–floc–Ar, the carbon coating layer relieved agglomeration, and the surface area of g–floc–Ar remained higher than that of g–floc–air.^[^ ^50^ ^]^

After the first cycle, the voltage–current curves almost overlapped; therefore, the electrodes obtained from microplastics through electrocoagulation with subsequent heat treatment established good structural integrity, leading to the stable and superior electrochemical reversibility of the sample.^[^ ^51^ ^]^

**Figure 6:**
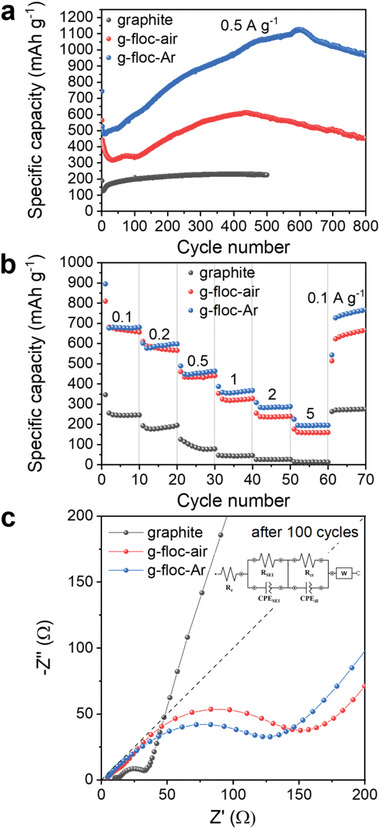
Electrochemical characterization data of each sample. a) Battery discharge profiles at a constant current density of 0.5 A g^−1^, b) rate capability test results at a rate from 0.1 to 5 A g^−1^, and c) electrochemical impedance spectroscopy (EIS) of graphite, g–floc–air, and g–floc–Ar electrodes.

**Figure** **6**a shows the cycling performance of graphite, g–floc–air, and g–floc–Ar at a current density of 0.5 A g^−1^. As expected, for graphite, a stable specific capacity of ≈225 mAh g^−1^ was maintained for 500 cycles. However, the g–floc–air and g–floc–Ar electrodes show distinctly different tendencies during long‐term cycling. The profile of the electrodes obtained from microplastics via electrocoagulation exhibited a gradual increase in capacity up to several hundred cycles, termed negative fading.^[^ ^52^, ^53^ ^]^ For g–floc–air, the specific capacity increased to 604 mAh g^−1^ at the 450th cycle, slightly higher than the theoretical capacity (562.5 mAh g^−1^), and suddenly collapsed. The lithium storage behavior of the g–floc–Ar electrodes is similar to that of g–floc–air, reflecting negative fading. The increase in the specific capacity of g–floc–Ar during the negative fading stage is considerably steeper (1123 mAh g^−1^ at the 450th cycle) than that of g–floc–air, resulting in a capacity more than twice the theoretical capacity (538.2 mAh g^−1^). Interestingly, the number of cycles that reached the deterioration stage was delayed from 450 to 600. This outcome implies that the amorphous carbon layer encapsulating the Fe_3_O_4_ nanoparticles relieved the volume expansion of the active materials and maintained the interface stably, inducing sufficient negative fading.^[^ ^51^, ^54^ ^]^ The volume expansion of the g–floc–Ar electrode was 16.2% after 2000 cycles at a current density of 5 A g^−1^, smaller than that of the g–floc–air electrode (19.8%) (Figure S11, Supporting Information).

The electrodes were evaluated at various current densities ranging from 0.1 to 5 A g^−1^ in the voltage range of 0.01–3 V (vs Li/Li^+^) without the activation cycles to investigate the rate performance of g–floc–air and g–floc–Ar (Figure 6b). The g–floc–Ar electrode exhibited a higher specific capacity than g–floc–air at higher current densities, demonstrating outstanding rate capabilities and structural stability. In addition, when the current density was returned to 0.1 A g^−1^ after the 60th cycle, a high specific capacity of the g–floc–Ar electrode was obtained, compared to the initial specific capacity, with a continuously increasing trend. The improved electrochemical performance, including the increased specific capacity with enhanced negative fading stage, delayed deterioration, and better rate performance, can be attributed to the introduction of an amorphous carbon layer derived from PE as microplastics suspended in the medium. This amorphous carbon layer is beneficial for reinforcing the electrical contact between the active material and the current collector and promoting the conduction of electrons in the electrode material. Electrochemical impedance spectroscopy (EIS) analyses of the graphite, g–floc–air, and g–floc–Ar electrodes after 100 cycles were performed further to demonstrate the improved conductivity and electron/ion transport. Detailed equivalent circuit fitting data are shown in Figure S12 and Table S3 (Supporting Information). As shown in Figure 6c, the semicircles in the high‐frequency region are caused by the charge‐transfer resistance (𝑅_𝑐𝑡_), indicating of the electrode–electrolyte interface while the diagonal line represents the diffusion process of lithium ions inside the material.^[^ ^55^ ^]^ Although the 𝑅_𝑐𝑡_ of g–floc–air (143.26 Ω) and g–floc–Ar (128.80 Ω) is larger than that of graphite (24.06 Ω) due to the poor electric conductivity of metal oxide, Fe_3_O_4_, 𝑅_𝑐𝑡_ of g–floc–Ar is smaller compared to that of 𝑅_𝑐𝑡_ of g–floc–air, which can result in improved reaction kinetics with lower resistance and a higher electrical conductivity.^[^ ^56^ ^]^

**Figure 7:**
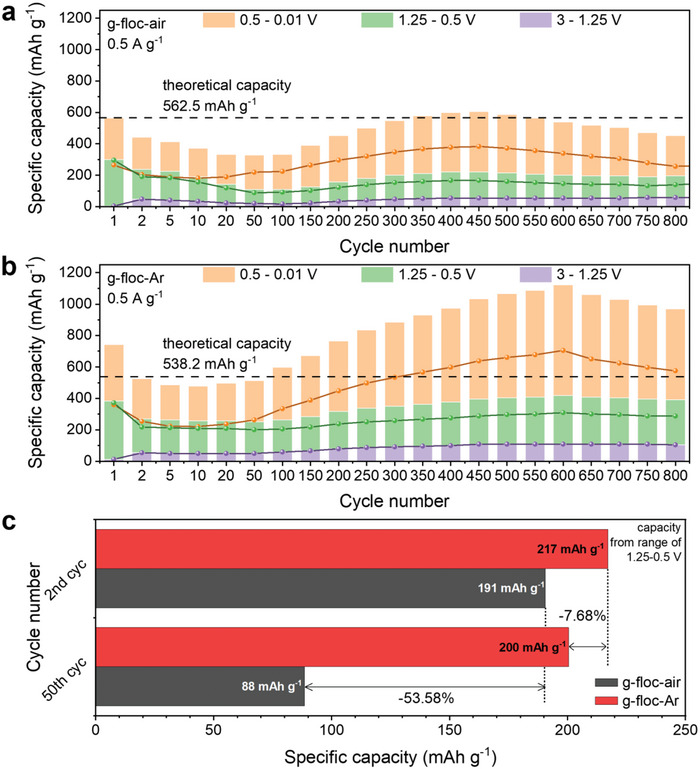
Capacity profiles divided into three voltage ranges of a) g–floc–air and b) g–floc–Ar electrodes. c) Comparison of iron oxide derived capacity of g–floc–air and g–floc–Ar electrodes in the voltage range of 1.25–0.5 V.

For iron oxide as an anode for LIBs, the insertion of Li ions into the active materials can be divided into different regions following the voltage range: insertion of Li ions into the crystal structure of iron oxides (1.25–3.0 V), conversion reaction of iron oxides (0.5–1.25 V), and reactions related to the electrolyte‐derived surface layer (0.01–0.5 V).^[^ ^52^ ^]^ The specific capacity at a selected cycle number is a function of the divided three regions (0.01–0.5, 0.5–1.25, and 1.25–3.0 V) (**Figure** **7**a,b). For the graphite electrode, most of the capacity was manifested by the intercalation and deintercalation of Li ions in the low‐voltage region (Figure S13, Supporting Information). The d*Q*/d*V* plots provide clues to further understanding the negative fading phenomenon. The cathodic and anodic peaks around 0.1 V in Figure S14a (Supporting Information) show that the capacity of graphite only came from the intercalation and deintercalation of Li in carbon material. On the other hand, Figure S14b,c (Supporting Information) featured the capacity appearing at the low potential range in addition to active material peaks. The increase in graph area in the low potential range during the battery cycling is related to the negative fading phenomenon. Furthermore, Figure S14c (Supporting Information) shows the larger graph area than g–floc–air, and those peaks remain relatively in the initial state after 800 cycles. This result corresponds to the fact that g–floc–Ar exhibits higher negative fading and cycle stability in the capacity profiles. Note that there is a tendency to match the long‐term cycle performance of the electrodes well with the specific capacity in the voltage range from 0.01 to 0.5 V (orange columns). In commercial LIBs using organic electrolytes, reduction of electrolyte occurs at the anode surface at low potential, resulting in the formation of electrolyte‐derived surface layer composed of thick polymeric/gel‐like film and thin SEI. Therefore, the negative fading resulting from specific capacity enhancement at low potentials is correlated with the electrolyte‐derived surface layer, which contributes to the structural and cyclic stability of the electrode. At present, the origin of the abnormal extra capacity in anode electrodes based on conversion reactions is generally recognized in the formation and decomposition of the electrolyte‐derived surface layer, in addition to the mechanism of the reoxidation and structural reorganization of the electrode material. This reaction occurs at an extremely low potential after completion of the conventional reaction in the bulk. The decomposition of the electrolyte‐derived surface layer is attributed to the catalytic process by forming small metallic nanoparticles formed after the conversion reaction (Equation (7)).^[^ ^53^ ^]^ Apart from the negative fading of the electrodes mainly originating from the formation and decomposition of the electrolyte‐derived surface layer at low potentials, after the second cycle, there was an anomalous degradation of capacity of ≈53.58% in the voltage range of 0.5–1.25 for g–floc–air electrode. Meanwhile, the capacity of the g–floc–Ar electrode remained constant, with only a 7.68% decrease (Figure 7c). This result indicates that the conversion reaction and degree of lithiation into the crystal structure of iron oxides in the g–floc–air electrode were reduced. This outcome was attributed to the detachment of the active materials owing to the pulverization of the electrode by intensive volume expansion during the charging/discharging process.^[^ ^57^ ^]^ However, for the g–floc–Ar electrode, the carbon shell surrounding the Fe_3_O_4_ nanoparticles mitigates the volume expansion to prevent the loss of the active material, thereby preserving the capacity originating from the conversion of iron oxide and lithiation into the crystal structure. Overall, the iron oxide core–carbon shell powder obtained via electrocoagulation with subsequent heat treatment in an Ar atmosphere can be applied as an anode material for LIBs. The powder has excellent electrochemical performance, simultaneously removing microplastics, which are pointed out as the main culprit of ecological destruction.

## 3 Conclusions

In summary, a strategy for electrocoagulation using Fe foil as a sacrificial electrode to effectively remove microplastics suspended in an aqueous medium was studied. Fe_3_O_4_ nanoparticles were transformed through the *γ*‐FeOOH intermediate from the initial Fe(OH)_2_ prepared by Fe^2+^‐eluted cations at the electrode. These nanoparticles were uniformly decorated on the surface of PE to destabilize suspended microplastics, resulting in agglomerated powders for easy separation using the magnetic properties of Fe_3_O_4_–PE flocs with high efficiency of 98.4%. It was revealed that PE microplastics of different sizes could be collected through electrocoagulation. When 3.5 wt% NaCl was used as a medium, the suspended microplastics effectively sediment even at low overpotentials. This implies the possibility of removing microplastics contained in seawater with low energy consumption if the effect of various sea salts on electrocoagulation is verified. We demonstrated that the Fe_3_O_4_ nanoparticles, which were revealed as inhibitors of the photodegradation of microplastics under UV irradiation, should be removed from the Fe_3_O_4_–PE flocs in oxalic acid before photolysis. In addition, the oxalic‐acid‐dissolved Fe_3_O_4_ can be electrochemically redeposited to Fe compounds on the substrate, which indicates that the proposed process can be developed into a zero‐pollution‐emission process or produce valuable resources for use as an anode material for next‐generation LIBs.

Fe_3_O_4_–PE flocs were employed as anode materials for LIBs after heat treatment. In the case of Fe_3_O_4_–PE flocs after heat treatment in an Ar atmosphere, an amorphous carbon layer with a uniform thickness of less than 2 nm derived from the PE is decorated on the surface of Fe_3_O_4_ nanoparticles, forming an oxide core–carbon shell structure. This result was attributed to forming a stable SEI layer, relief of the volume expansion of Fe_3_O_4_, and high conductivity between the active materials. A remarkable capacity increase is observed in the evaluation of the long‐term cycling stability of the electrodes; in particular, g–floc–Ar shows a more rapid capacity increase and stable cycling characteristics with a low volume expansion after 2000 cycles at a current density of 5 A g^−1^, compared to g–floc–air. Because the increase in capacity generally corresponds to the capacity component in the low‐voltage region, it is concluded that the negative fading is caused by the formation and decomposition of the electrolyte‐derived surface layer, which leads to the structural stability of the electrode. This study provides an environmentally sustainable strategy for preparing Fe_3_O_4_ core–carbon shells as promising anodes for LIBs from microplastics suspended in an aqueous medium, suggesting a way to simultaneously address environmental and energy issues.

## Conflict of Interest

The authors declare no conflict of interest.

## Author Contributions

J.L. performed the experiments and wrote the paper. Y.‐T.K. advised on the experiment and wrote the paper. J.C. supervised the research and revised the paper.

## Supporting information

Supporting Information

Supplemental Video 1

## Data Availability

The data that support the findings of this study are available from the corresponding author upon reasonable request.
