# Supplementary material for: Recycling Microplastics to Fabricate Anodes for Lithium‐Ion Batteries: From Removal of Environmental Troubles via Electrocoagulation to Useful Resources
Source: Adv Sci (Weinh). 2023 Jan 16;10(8):2205675. doi: 10.1002/advs.202205675 (PMC10015874; doi:10.1002/advs.202205675)
Supplement: Supplementary file 1 — Supporting Information [file ADVS-10-2205675-s001.pdf]

Supporting Information

Recycles of Microplastics for Fabrication of Anode in Lithium-Ion Battery: from Removal of Environmental Troubles via Electrocoagulation to Useful Resources

Jinhee Lee, Yong-Tae Kim, Jinsub Choi**

Department of Chemistry and Chemical Engineering

Inha University, 22212, Incheon, Republic of Korea

*To whom correspondence should be addressed.

Tel: +82-32-860-7476

e-mail: yongtaekim@inha.ac.kr, jinsub@inha.ac.kr

Table of contents

Experimental Procedures

Materials

Electrocoagulation of polyethylene

Photodegradation of polyethylene

Material characterizations

Electrochemical measurements

Supporting Figures and Tables

**Figures S1-S4**	Electrocoagulation of PE.
**Figures S5-S6, and Table S1.**	Photodegradation of Fe_3O_4–PE flocs.
**Figures S7-S14, and Table S3.**	Application of flocs as anode material for lithium-ion battery.

Experimental Procedures

Materials

Polyethylene powder (PE, 40 µm or 200 µm, Sigma-Aldrich) was used as a representative material for microplastics. All chemicals, including sodium chloride (NaCl, 99.8%, Sigma-Aldrich), oxalic acid ($C_2H_2O_4$, 98%, Sigma-Aldrich), iron foil (0.125 mm, 99.5%, GoodFellow), copper foil (0.127 mm, 99.9%, Alfa Aesar/9 µm, 99.99%, MTI Corp.), graphite

powder (<20 µm, synthetic, Sigma-Aldrich), poly(vinylidene fluoride) (PVDF, average Mw 534,000 powder, Sigma-Aldrich), carbon black (Super P, >99%, Alfa Aesar), and N-methyl-2-pyrrolidone (NMP, anhydrous, 99.5%, Sigma-Aldrich) were purchased from commercial suppliers and were used directly without further purification.

Electrocoagulation of polyethylene

No unexpected or unusually high safety hazards were encountered during all experiments described below. Two iron foils (1.5 cm × 3.3 cm) placed about 4 cm apart were pretreated by sonicating in acetone and ethanol for 10 min, respectively. A NaCl solution (200 mL) containing 0.5 g of PE powder was employed in the electrocoagulation process. For comparison, the PE size and NaCl concentration were adjusted between 40 and 200 µm and 0.4 and 3.5 wt.%, respectively. A suspension of Fe_3O_4–PE flocs were obtained at a constant current density of 5 mA cm^{-2} by stirring at 800 rpm for 30 min. Fe_3O_4–PE flocs were completely separated from the solution by applying external magnetic fields. After discarding the supernatant, the flocs were washed with deionized water for further characterization. Finally, the magnetic-field-adsorbed flocs were dried at 60 °C for 24 h to measure their

weight. The PE removal efficiency was calculated using Equation (1):

$$Removal\ efficiency\ (\%) = \frac{(Fe_3O_4 - PE)_{floc} - (Fe_3O_4)_{floc}}{PE_0} \times 100 \quad (1)$$

where PE_0 is the initial amount of PE (0.5 g), $(Fe_3O_4–PE)_{floc}$ is the weight of Fe_3O_4–PE flocs, and $(Fe_3O_4)_{floc}$ is the weight of Fe_3O_4 flocs obtained without the addition of PE to the initial solution.

Photodegradation of polyethylene

Photodegradation was performed by irradiating the samples with four UV–C lamps (8 W, 254 nm) for 300 h. The distance between the samples and the UV–C lamps was maintained at 5 cm using a homemade rectangular box. The weight of each sample was measured every 24 h during photodegradation. The carbonyl index (CI), defined as the ratio of the band area attributed to the carbonyl group (C=O) to the band area attributed to the methylene group (CH_2), was used to evaluate the oxidation level of PE. The CI confirmed the degradation of the samples based on the ratio between the integrated area of 1,850–1,650 cm^{-1} and 1,500–1,420 cm^{-1} from the Fourier Transform Infrared Spectroscopy (FT-IR) data, as shown in Equation (2).

$$CI = \frac{Integrated\ area\ of\ 1{,}850-1{,}650\ cm^{-1}}{Integrated\ area\ of\ 1{,}500-1{,}420\ cm^{-1}} \quad (2)$$

Material characterizations

The morphologies of PE and floc were identified by field-emission scanning electron

microscopy (FE-SEM; S-4300, Hitachi), combined with energy-dispersive X-ray spectroscopy (EDX) and field-emission transmission electron microscopy (FE-TEM; JEM-2100F, JEOL), at the Core Facility Center for Sustainable Energy Materials of Korea Basic Science Institute (KBSI). The crystallinity of the samples was analyzed by X-ray diffraction (XRD; X'Pert powder diffractometer, Malvern Panalytical) with CuK_{α} radiation. The functional groups were determined by Fourier-transform infrared (FT-IR) spectroscopy (VERTEX 80v, Bruker). The chemical states were measured using X-ray photoelectron spectroscopy (XPS; K–Alpha, Thermo Fisher Scientific). Thermogravimetric analysis (TGA; TG 209 F3 Tarsus, Netzsch) was performed at 800 °C at a ramping rate of 5 °C min^{-1} with air or argon flow.

Electrochemical measurements

Before electrochemical analysis, Fe_3O_4–PE flocs were annealed at 500 °C with a ramping rate of 5 °C min^{-1} for 2 h under air or Ar atmospheres. Then, the heat-treated floc and graphite were mixed in a weight ratio of 3:7 to prepare an active material denoted as g–floc–air and g–floc–Ar, respectively. The as-prepared active material, conductive agent (Super P), and poly(vinylidene fluoride) (PVDF) binder were mixed at a weight ratio of 8:1:1 and dispersed in NMP to form a slurry. The obtained slurry was homogeneously coated onto a Cu foil current collector and dried in a vacuum oven at 120 °C for 12 h. A disk with a diameter of 14 mm was punched out and used as the working electrode. The areal mass loading of prepared electrodes was 3.5 mg cm^{-2}. Electrochemical measurements were performed in a half–cell configuration using CR2032-type coin cells assembled in an Ar–filled glove box that maintained an oxygen content below 0.1 ppm. Metallic Li, Celgard 2400, and 1 M $LiPF_6$ in

ethylene carbonate (EC)/diethylene carbonate (DEC) (1:1 v/v) were used as the counter electrode, separator, and electrolyte, respectively. Galvanostatic charge/discharge cycle performance was evaluated using a battery cycler system (WBCS3000S1, WonATech) at current densities of 0.1–5 A g^{-1}. Electrochemical impedance spectroscopy (EIS) was conducted on an electrochemical workstation (Autolab PGSTAT128N, Metrohm) in the frequency range from 100 kHz to 0.01 Hz.

Supporting Figures and Tables

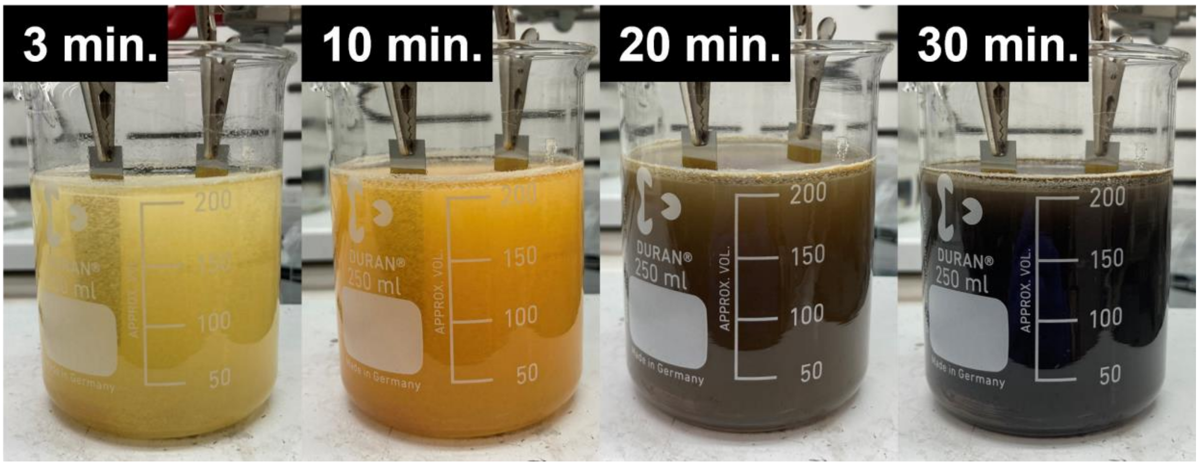

Figure S1. Photographs showing the change in color of the electrolyte during 30 min of EC reaction.

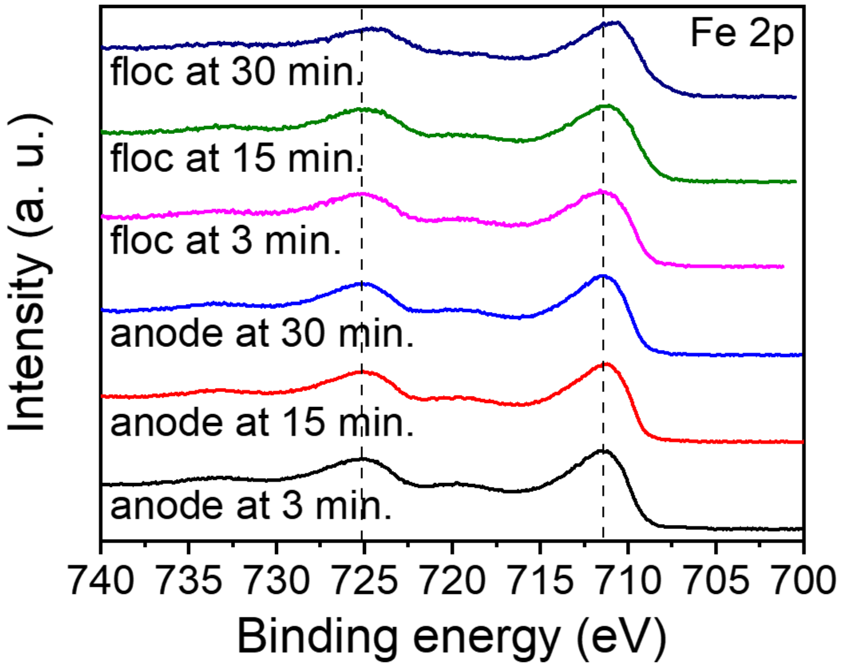

Figure S2. X-ray photoelectron spectroscopy (XPS) Fe 2p spectra of anode surface and collected flocs in different reaction times. The dotted line is the peak of the anode at 3 min.

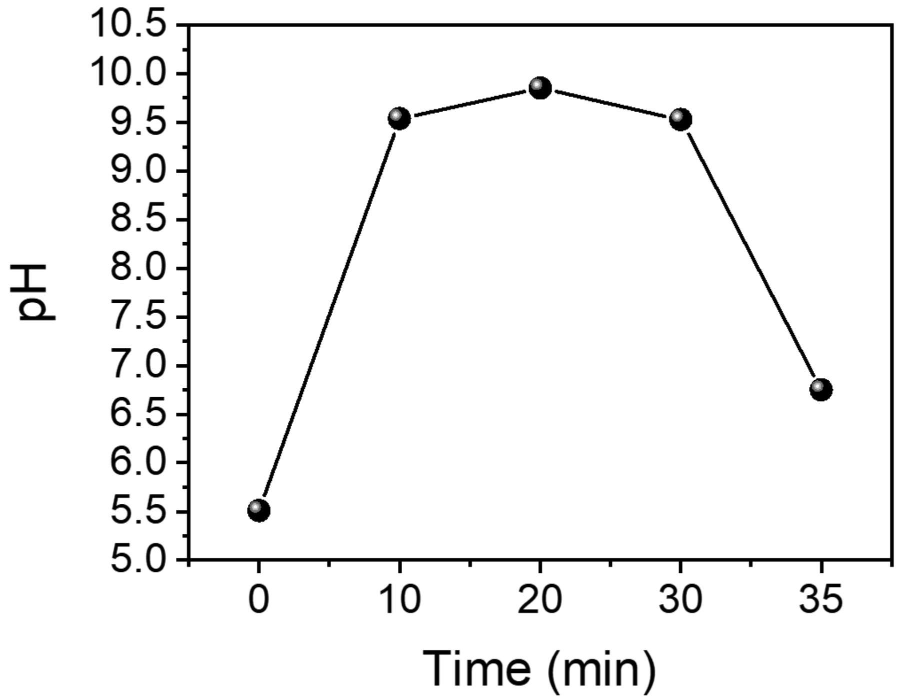

Figure S3. The pH change profile of NaCl solution during EC reaction time. The datum at 35 min is gained 5 minutes after the end of EC reaction with stirring, but no electrical apply.

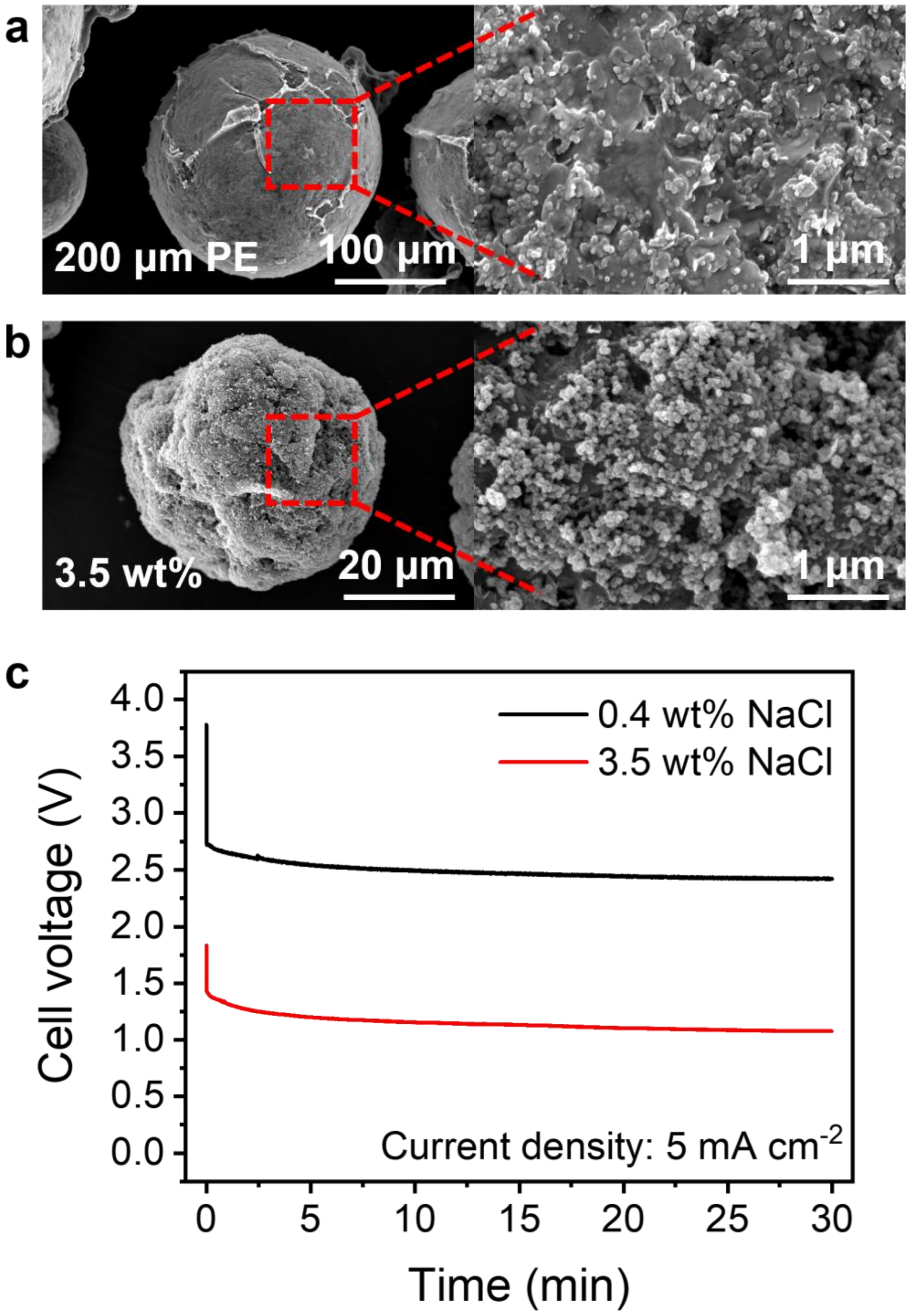

Figure S4. Scanning electron microscopy (SEM) images with the conditions of (a) 200 μm sized PE and 0.4 wt% NaCl solution, and (b) 40 μm sized PE and 3.5 wt% NaCl solution. (c) Voltage-time profiles during EC reaction. Black line data is collected in 0.4 wt% of NaCl solution, and red line data is acquired in 3.5 wt% of NaCl solution. All other experimental conditions remained the same.

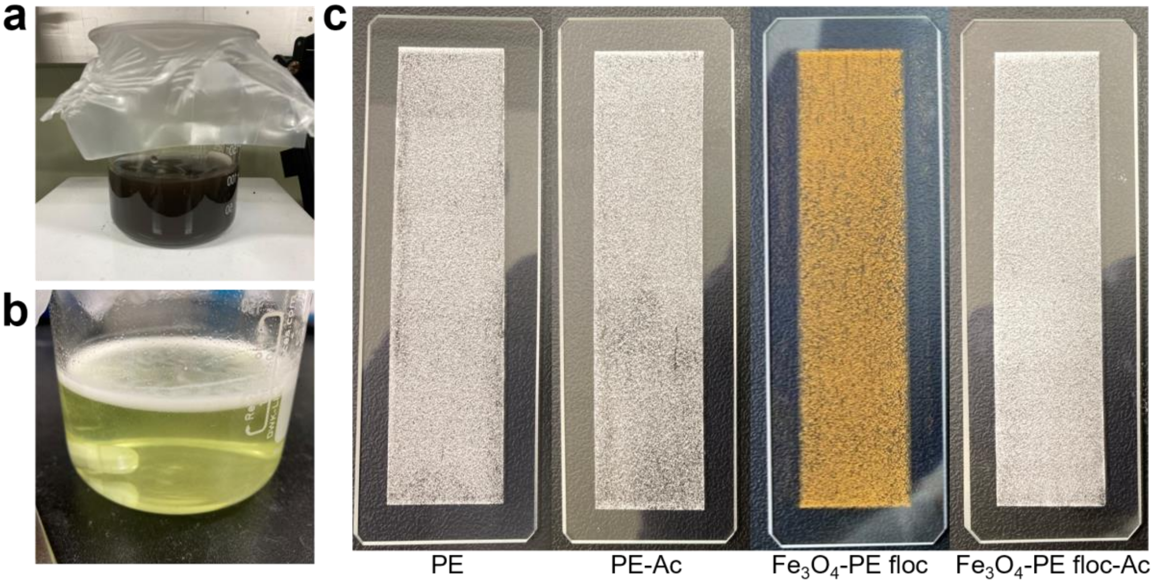

Figure S5. Photographs of iron oxide dissolving process. (a) EC floc stirred in 0.3 M oxalic acid, (b) iron dissolved solution after 1 h stirring. (c) Photograph of photodegradation testing sample.

Table S1. Carbonyl index calculated from FT-IR data before and after 300 h photodegradation.

Carbonyl index	PE	PE-Ac	Fe_3O_4-PE floc-Ac	Fe_3O_4-PE floc
**0 h**	0.098	0.094	0.091	0.141
**300 h**	1.398	1.634	1.482	0.413

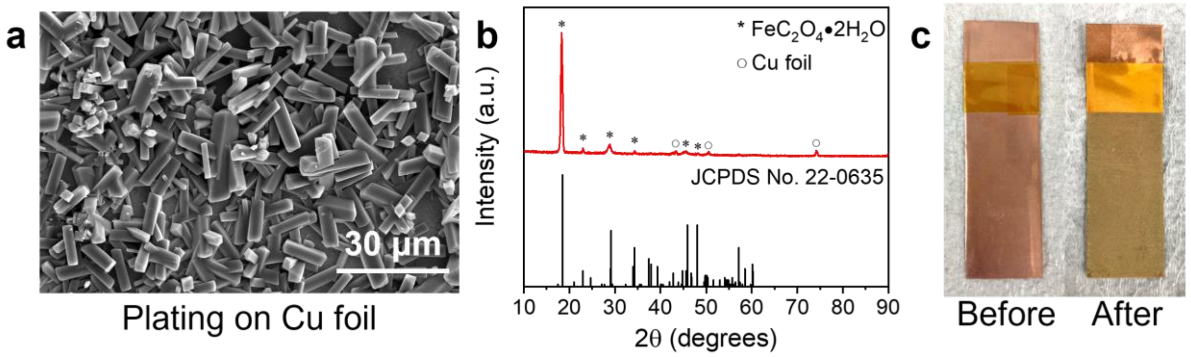

Figure S6. (a) Scanning electron microscopy (SEM) image of copper foil after electrodeposition. The solution in Fig. S1b was used as the electrolyte for deposition. (b) X-ray diffraction (XRD) spectrum of copper foil after electrodeposition. (c) Photographs of copper foil before and after electroplating.

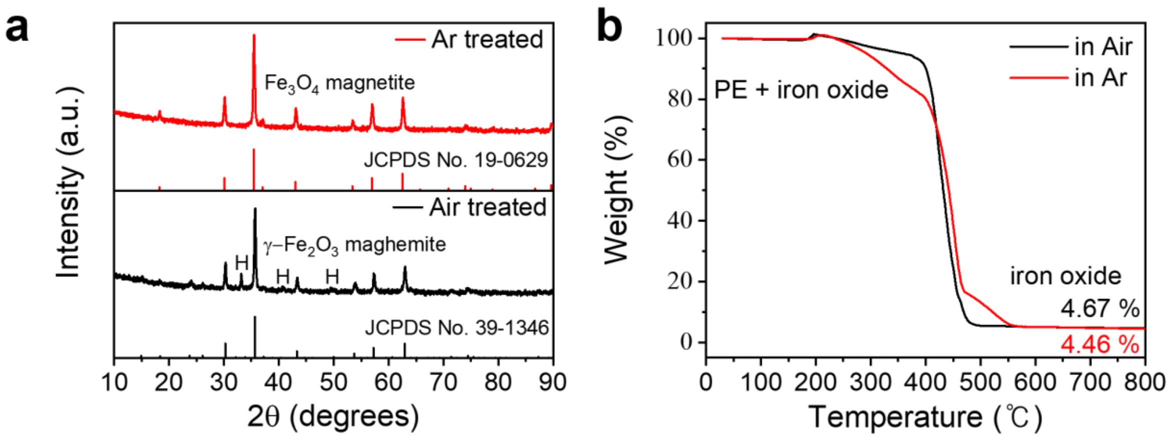

Figure S7. (a) X-ray diffraction (XRD) spectra of the heat-treated floc samples in air or argon atmosphere. (b) Thermogravimetry analysis (TGA) data of EC floc in air atmosphere.

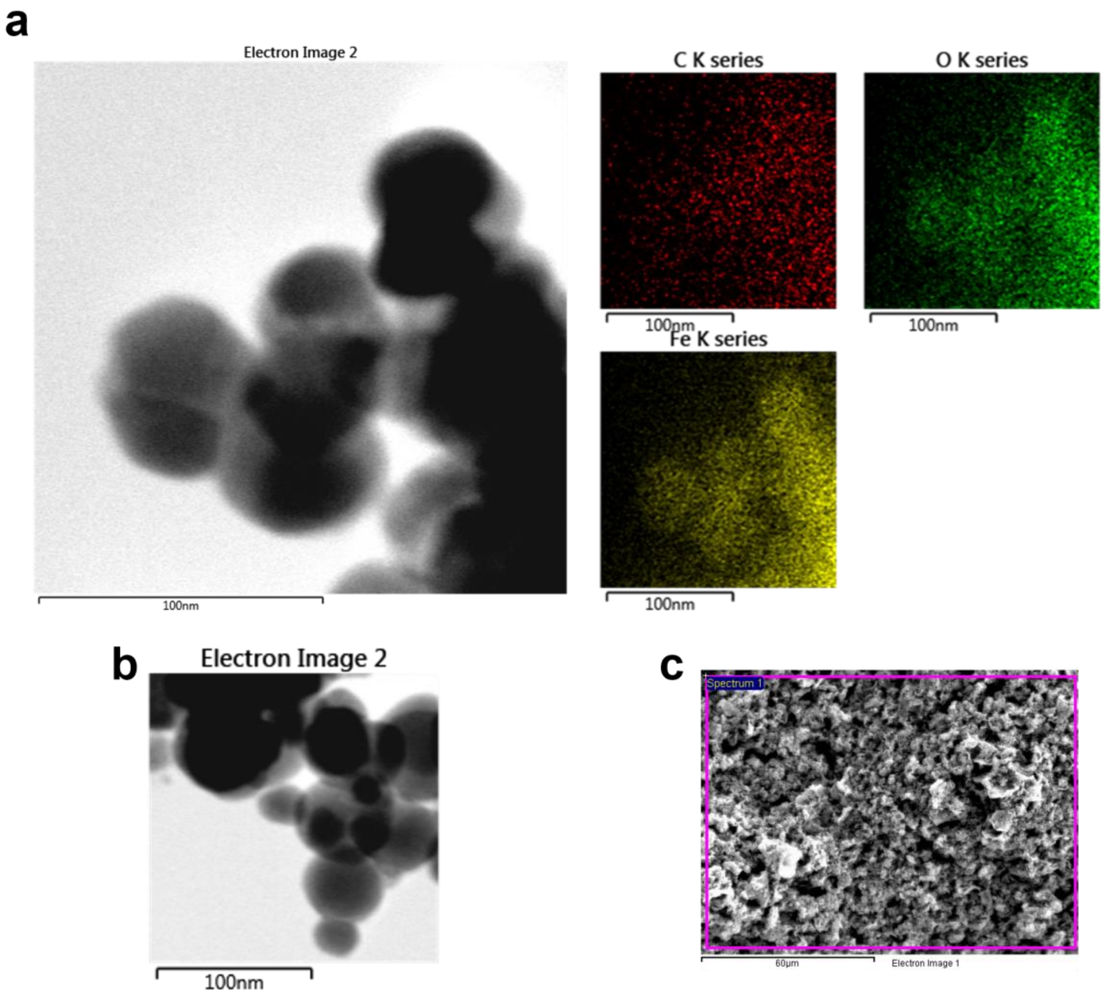

Element	Wt%	Wt% Sigma	Atomic %
C	7.94	0.33	17.69
O	31.96	0.30	53.50
Fe	60.10	0.33	28.81
Total:	100.00		100.00

Element	Wt%	Wt% Sigma	Atomic %
C	7.87	1.41	17.68
O	31.44	0.77	53.01
Fe	60.69	1.10	29.32
Total:	100.00		100.00

Figure S8. (a) The elemental mapping images of transmission electron microscopy (TEM) of heat-treated floc at 500 °C with argon flow. (b) Elemental compositions analyzed by TEM-EDX, and (c) SEM-EDX

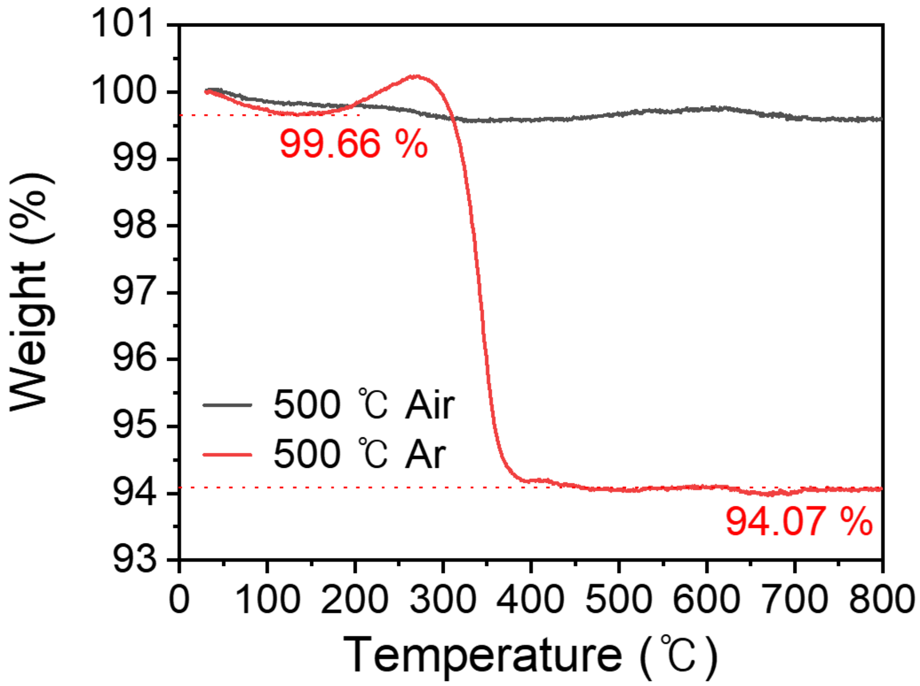

Figure S9. Thermogravimetry analysis (TGA) data of heat-treated floc. TGA measurements were conducted with air flow. The weight ratio of Fe_3O_4:C of the heat-treated sample in Ar was calculated from the following sequences.

Basis 100 g	(Fe_3O_4 + C + H_2O)
99.66 g	(Fe_3O_4 + C)
94.07 g	(Fe_2O_3)
159.69 g/mol	(Fe_2O_3)
0.59 mol	(Fe_2O_3)
1.18 mol	(Fe)
0.39 mol	(Fe_3O_4)
231.53 g/mol	(Fe_3O_4)
90.97 g	(Fe_3O_4)
8.69 g	(C)
10.47 : 1	(Fe_3O_4 : C)

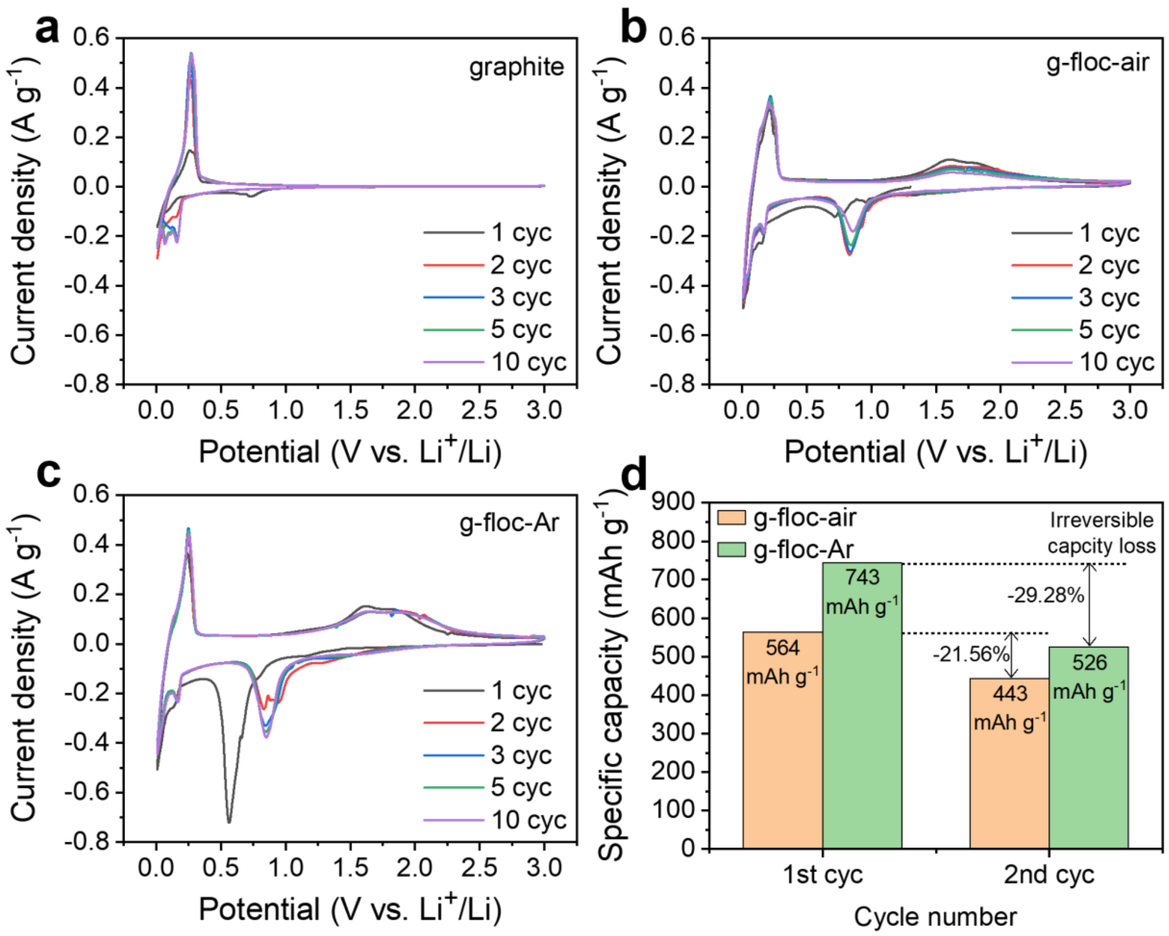

Figure S10. Cyclic voltammetry curves of (a) graphite, (b) g-floc-air, and (c) g-floc-Ar electrodes. (d) Irreversible capacity loss of g-floc-air and g-floc-Ar electrodes between 1st and 2nd cycle.

Table S2. BET surface areas of g-floc-air and g-floc-Ar nanoparticles.

	g-floc-air	g-floc-Ar
**BET surface area (m^2 g^{-1})**	19.5409	26.6523

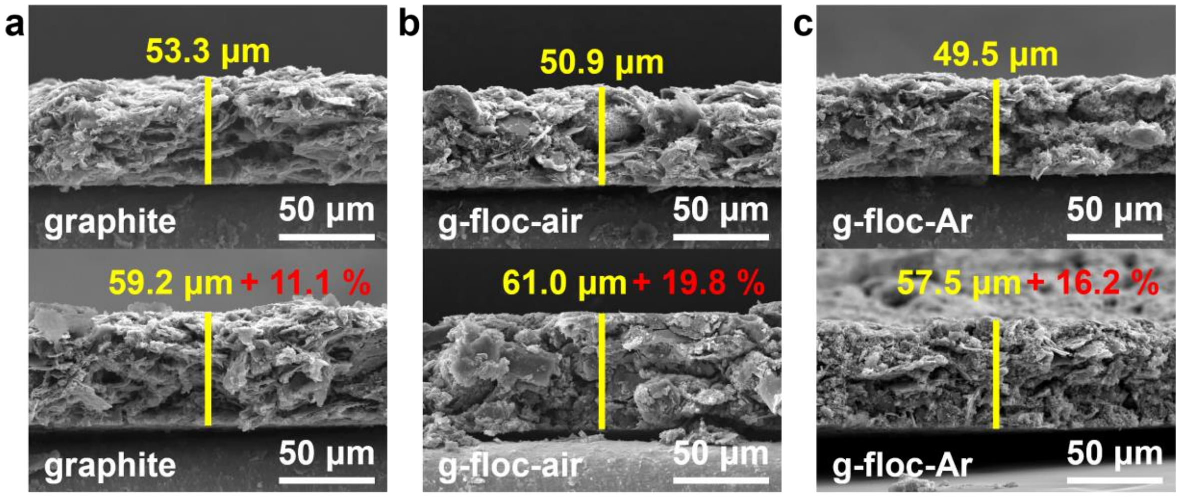

Figure S11. Scanning electron microscopy (SEM) cross-sectional images before and after 2000 cycles at a current density of 5 A g^{-1}. (a) graphite, (b) g-floc-air, and (c) g-floc-Ar.

Figure S12. Electrochemical impedance spectroscopy (EIS) with equivalent circuit fitting data of (a) graphite, (b) g–floc–air, and (c) g-floc-Ar electrodes.

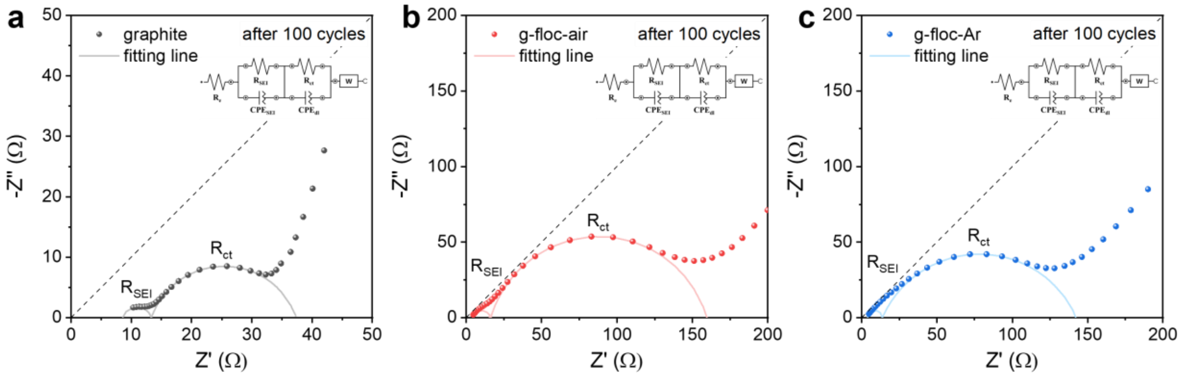

Table S3. Resistance values calculated by equivalent circuit fitting of EIS data.

	**graphite**	**g-floc-air**	**g-floc-Ar**
**R_s (Ω)**	8.56	3.86	4.06
**R_{SEI} (Ω)**	4.78	12.51	9.70
**R_{ct} (Ω)**	24.06	143.26	128.80

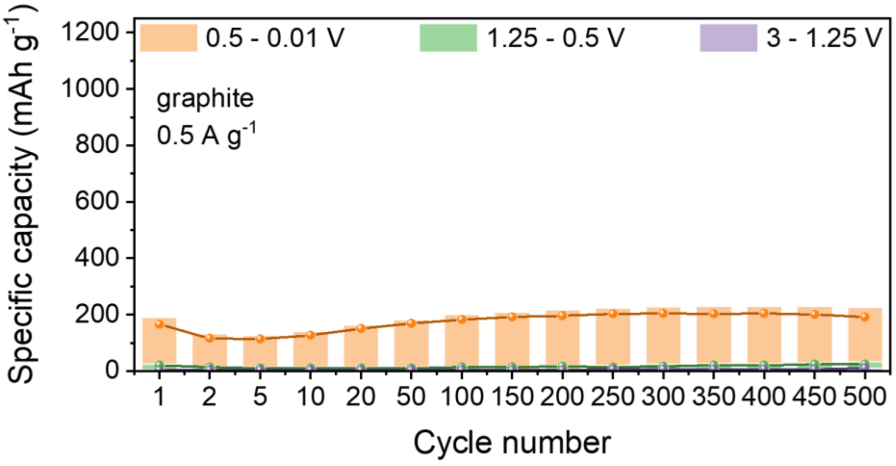

Figure S13. Capacity profiles of graphite electrode collected from three voltage ranges.

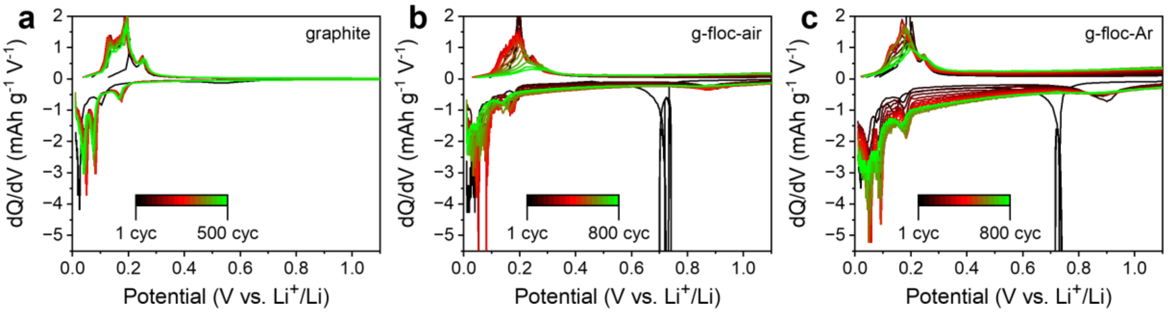

Figure S14. dQ/dV plots for (a) graphite, (b) g-floc-air, and (c) g-floc-Ar obtained from capacity profiles in Figure 6(a).
